# Retinal Neurodegeneration in Diabetes: an Emerging Concept in Diabetic Retinopathy

**DOI:** 10.1007/s11892-021-01428-x

**Published:** 2021-12-13

**Authors:** Mira M. Sachdeva

**Affiliations:** Wilmer Eye Institute, Johns Hopkins University School of Medicine, 600 North Wolfe Street, Maumenee 748, Baltimore, MD 21287 MD USA

**Keywords:** Diabetic retinopathy (DR), Neurodegeneration, Diabetes

## Abstract

**Purpose of Review:**

Diabetic retinopathy (DR), the leading cause of blindness in working-aged adults, remains clinically defined and staged by its vascular manifestations. However, early retinal neurodegeneration may precede vascular pathology, suggesting that this neuronal damage may contribute to disease pathogenesis and represent an independent target for intervention. This review will discuss the evidence and implications for diabetic retinal neurodegeneration.

**Recent Findings:**

A growing body of literature has identified progressive retinal thinning and visual dysfunction in patients with diabetes even prior to the onset of DR, though advances in retinal vascular imaging suggest that vascular remodeling and choroidal changes occur during these early stages as well. Animal models of diabetes and in vitro studies have also suggested that diabetes may directly affect the retinal neural and glial tissue, providing support to the concept that diabetic retinal neurodegeneration occurs early in the disease and suggesting potentially relevant molecular pathways.

**Summary:**

Diabetic retinal neurodegeneration may represent a “preclinical” manifestation of diabetic retinal disease and remains an active area of investigation. As the natural history and molecular mechanisms become increasingly understood, it may lead to upcoming developments in not only the treatment options but also the clinical definition of DR.

## Introduction

Diabetes mellitus (DM) remains a growing epidemic worldwide with significant morbidity and mortality, and diabetic retinopathy (DR) represents the leading cause of blindness in the working-age population [1]. DR has long been considered a disease of the retinal microvasculature and indeed is still diagnosed and staged by microvascular manifestations evident on clinical examination. These findings range from the microaneurysms, cotton-wool spots, intraretinal hemorrhages, venous beading, and intraretinal microvascular abnormalities that define the spectrum of nonproliferative DR to the extensive ischemia, neovascularization, and subsequent potential vitreous hemorrhage and tractional retinal detachments that define more advanced proliferative DR. DR stage guides treatment recommendations and monitoring intervals due to increasing risk of severe vision loss as the disease progresses. A recent study using the American Academy of Ophthalmology’s Intelligent Research in Sight (IRIS) registry demonstrated that in eyes with good initial vision, severity of DR at first diagnosis was a risk factor for the development of blindness, reflecting the persistent clinical utility of this classification scheme [2]. However, during the past several years, an increasing body of evidence suggests that retinal neurodegeneration represents an additional component of diabetic retinal disease that may precede the retinal vascular abnormalities [3]. Understanding the onset, pathogenesis, and progression of these “preclinical” manifestations of DR may uncover novel strategies for earlier detection and treatment. This review will discuss the evidence for early diabetic retinal neurodegeneration from patients as well as experimental models, suggest potential molecular mechanisms and relevant cell types involved, and highlight the implications for the diagnosis and treatment of diabetic retinal disease.

## Evidence for Retinal Neurodegeneration in Pre-clinical DR

While the diagnostic criteria for DR were developed at a time when clinical exam, color fundus photographs, and fluorescein angiography were the mainstays of retinal evaluation, the introduction of optical coherence tomography (OCT) imaging to noninvasively and rapidly visualize the layers of the macula in cross-section has revealed alterations in retinal structure prior to the onset of DR. To date, many studies have now used OCT to demonstrate thinning of the inner retina including the retinal nerve fiber layer (RNFL), ganglion cell layer (GCL), and more variably the inner plexiform layer (IPL) in patients with type 1 or type 2 DM without DR [4, 5]. These structural changes are not limited to the macula. Decreased peripapillary RNFL thickness has been described in diabetic patients with or without DR, with RNFL thickness inversely associated with glycated hemoglobin (HbA1c), duration of diabetes, and severity of DR [6, 7].

In addition to inner retinal thinning, functional deficits in contrast sensitivity, perimetry testing, multifocal electroretinogram (mfERG), and dark adaptation have also been described in diabetic patients without DR or with very early DR [8–13]. A correlation between GCL thinning and visual field deficits on Rarebit perimetry was demonstrated in type 1 DM patients with no DR [14]. Similarly, a recent large cohort study of diabetic patients without DR demonstrated an association between functional deficits on frequency doubling technology (FDT) perimetry or mesopic microperimetry and retinal ganglion cell count, as inferred from GCL thickness on OCT [15]. However, other concurrent assessments of microperimetry and OCT suggest a more complex relationship between inner retinal structure and retinal function. Montesano et al. found differential structure/function relationships in patients with DM but no DR compared with controls [16]. Similarly, while Park et al. demonstrated decreased sensitivity on microperimetry as well as thinning of the inner retina (defined as the GCL/IPL/inner nuclear layer complex in this study) in type 2 diabetic patients with early or no DR, these functional and structural deficits were only weakly associated, suggesting that additional factors other than retinal thickness may contribute to early diabetic retinal dysfunction [17]. For example, disorganization of the inner retinal layers (DRIL) may contribute independently to visual field and contrast sensitivity deficits in patients with diabetes [18].

Interestingly, neuronal dysfunction in early diabetic retinal disease may contribute to subsequent DR development and progression. Focal areas of delayed implicit time on mfERG have been associated with an increased risk of DR development at 1 year [19]. Progressive RNFL and GCL thinning in patients with type 1 DM without DR has been associated with the presence of DR at follow-up over a 6-year period [20••].

In summary, changes in inner retinal thickness and visual function deficits occur prior to clinical DR in patients with DM, suggesting a neurodegenerative component of diabetic retinal disease that may be independent from, or at least concurrent with, the vascular manifestations.

## Animal Models of Early Diabetic Retinal Neurodegeneration

The early inner retinal neurodegeneration evident in patients with type 1 and type 2 DM has been recapitulated in several animal models, emphasizing the evolutionary conservation of the retinal response to diabetic injury and providing systems within which to study these effects. Thinning of the GCL/IPL on OCT, and in some studies the inner nuclear layer (INL), outer nuclear layer (ONL), and total retina as well, has been described in both mice and rats treated with the pancreatic beta-cell toxin streptozotocin (STZ) to induce diabetes [21–24]. STZ-treated mice also demonstrate deficits in spatial frequency threshold and contrast sensitivity at 2 months [25]. However, frank RGC loss has been more consistently described in the rat model, with variable assessments of RGC number in STZ-treated mice [24, 26–28]. Of note, differences in glial activation have also been noted in STZ-treated rats compared with STZ-treated mice and suggest, with the STZ-induced model of diabetes, that the rat may more closely resemble changes seen in human diabetic retina [29]. Genetic mouse models of type 1 DM have also demonstrated inner retinal neuronal loss. For example, the Ins2^Akita^ mouse, which harbors a point mutation in the Insulin 2 gene leading to beta-cell death and subsequent diabetes, develops thinning of the IPL and INL by 22 weeks of hyperglycemia and reduced RGC number in the peripheral retina by 3 months [30, 31]. RGC apoptosis has also been described in non-obese diabetic (NOD) mice, a spontaneous model of autoimmune diabetes [32].

Several genetic and induced mouse models of type 2 DM demonstrate inner retinal thinning on OCT and/or retinal function deficits as well. Mice fed a high-fat diet exhibit impairments in a-wave, b-wave, and oscillatory potentials (OPs) on ERG testing, suggesting neuronal injury, though inner retinal thinning was not noted [33–35]. On the other hand, the db/db leptin-receptor-deficient mouse manifests both retinal functional deficits on ERG as well as RGC loss as early as 8 weeks of age [36–38]. Finally, diabetic retinal neurodegeneration has been documented, though less well-studied, in other animal models. For example, the sand rat, *Psammomys obesus*, can develop diabetes similar to type 2 DM that has been associated with retinal thinning and vascular changes [39].

## Molecular Mechanisms of Diabetic Retinal Neurodegeneration

Data derived from the various animal models described above, in vitro studies, and, perhaps most relevant, from the ocular fluids of diabetic patients have uncovered various molecular pathways that may mediate retinal neuronal damage in diabetes. Several of these have long been broadly implicated in DR pathogenesis but more recent evidence ties these pathways more specifically to retinal neurodegeneration.

### Metabolic Factors

Hyperglycemia itself can trigger cellular damage via protein kinase C (PKC) activation, oxidative stress, increased flux through the polyol pathway in which glucose is converted to sorbitol by aldose reductase, and generation of advanced glycation end-products (AGEs). Wang et al. demonstrated mitochondrial swelling in RGCs and decreased superoxide dismutase (SOD) activity in the retina of STZ-treated rats 4 weeks after induction of hyperglycemia, suggesting that oxidative stress may contribute to RGC loss [40]. Accumulation of AGEs and upregulation of the receptor for AGEs (RAGE) occurs in the diabetic retina and pharmacological inhibition of RAGE-mediated signaling pathways was shown to improve diabetes-associated abnormalities in implicit times in the b-wave and OPs at 6 months in the apoE^−/−^ db/db mouse [41]. Data from rodent studies, however, must be evaluated with caution as some species-dependent differences in molecular mechanisms of disease may exist. For example, a direct comparison demonstrated higher aldose reductase activity and increased sorbitol production in the retinas of STZ-treated diabetic rats compared with STZ-treated diabetic mice, accompanying phenotypic variations [42]. Despite this, mice deficient in aldose reductase are protected from impairments in contrast sensitivity and spatial frequency threshold after STZ treatment, supporting a role for increased sorbitol production in diabetic retinal neuronal injury [25]. Finally, insulin signaling itself via the Akt kinases regulates neuronal survival and retinal insulin resistance may lead to loss of this neurotrophic support. Fort et al. demonstrated that subconjunctival insulin administration partially rescued RGC death, with increased Akt1 activity and changes in inflammatory pathways, in the STZ-treated rat 4 weeks after induction of diabetes without reducing systemic hyperglycemia [43].

### Inflammation

The pro-inflammatory systemic milieu of DM is now well-recognized and increasing evidence suggests a pathologic role for diabetes-related inflammation within the retina. Microglial activation and increased expression of the inflammatory cytokines TNF-alpha and IL-1beta has been demonstrated in the retina of STZ-treated rats, and activated microglia produce factors that can induce retinal neuronal death in cell culture [44]. Microglial activation has also been identified by histopathologic analyses in post-mortem retinal specimens of patients with different stages of DR [45]. A recent study demonstrated that STZ-induced diabetes exacerbates RGC dysfunction and loss in the DBA/2 J mouse model of glaucoma, with an associated increased expression of oxidative stress markers and IL-6 and TNF-alpha [46]. Finally, several studies have identified elevated levels of inflammatory cytokines including TNF-alpha, IL-6, IL-1beta, and MCP in the aqueous or vitreous of patients with various stages of DR, further implicating inflammation in the pathogenesis of diabetic retinal disease [47–51].

### Potential Parallels with Neurodegenerative Disease

Reduced thickness of the RNFL, GCL, and GC/IPL complex on OCT in diabetic patients has been associated with diabetic peripheral neuropathy, suggesting that underlying molecular mechanisms of neuronal damage may be similar [52, 53]. Especially given the increasing data describing inner retinal thinning on OCT in patients with Alzheimer’s disease and Parkinson’s disease (PD), it is intriguing to consider potential parallels with molecular mechanisms known to play a role in neurodegenerative diseases [54, 55]. One potential common etiology may be glutamate excitotoxicity, which can induce cell death in post-synaptic neurons by several molecular pathways due to increased cellular calcium influx. Increased retinal glutamate levels have been described in the STZ-treated rat after 3 months of hyperglycemia, likely due to reduced expression of the glutamate transporter and resulting impaired glutamate uptake by Muller cells as well reduced glutamate conversion to glutamine by the Muller glia [56, 57]. Elevated glutamate levels have been found in the vitreous of patients with PDR [58]. Regarding other potential parallels with neurodegenerative diseases, Zhu et al. identified increased levels of phosphorylated tau protein in the retina of HFD-fed mice at 16–20 weeks, temporally correlating with dysregulation of synaptic proteins [59]. Their data from both the HFD model and primary RGCs in culture implicate increased signaling via the IRS-1/Akt/GSK3b pathway in this early diabetic neuronal dysfunction [59]. Additionally, increased immunostaining of PINK1 and parkin, key players in a pathway mediating mitochondrial dynamics well studied in the pathogenesis of PD, was identified in the RGCs of STZ-treated diabetic rats [60].

## Therapeutic Strategies Targeting Retinal Neurodegeneration in Diabetes

### Targeting Aberrant Molecular Pathways

Pharmacologic targeting of the pathophysiologic molecular pathways and mechanisms described above has been able to rescue diabetic RGC death and neuronal dysfunction in rodent models. As described above, subconjunctival insulin administration partially rescued RGC death in the STZ-treated rat model [43]. Additionally, administration of an exogenous ROS scavenger to STZ-treated rats rescued RGC mitochondrial abnormalities and ERG deficits [40].

### Neuroprotective Factors

An alternative therapeutic strategy involves enhancement of neuroprotective mechanisms that are impaired in the diabetic retina. Protein and mRNA levels of brain-derived neurotrophic factor (BDNF) are reduced in the retina of STZ-treated rats and aqueous levels of BDNF are decreased in eyes of diabetic patients even without DR compared with nondiabetic controls, suggesting a physiologically relevant role for this neurotrophic factor [61, 62]. Indeed, intravitreal adenoviral delivery of exogenous BDNF rescued RGC death in the STZ rat model [63]. Nerve growth factor (NGF) administration was also shown to reduce RGC apoptosis in the STZ-treated rat as assessed 15 weeks after diabetes onset [64]. Exogenous pigment epithelium-derived growth factor (PEDF) peptide delivered topically was shown to improve RGC survival in the Ins2^Akita^ mouse [65]. Finally, topical administration of ciliary neurotrophic factor (CNTF) eye drops rescued both b-wave amplitude deficits and inner retinal atrophy in the STZ-treated rat [66]. Growth factor signaling through other pathways may play a role in diabetic retinal neuroprotection as well. For example, high glucose-induced apoptosis of primary rat RGCs is inhibited by activation of the Notch/Jagged/HES pathway [67]. While none of the neurotrophic factors described above has been tested in diabetic patients, the prospective randomized-controlled EUROCONDOR trial assessed the efficacy of topical somatostatin or brimonidine in slowing or preventing retinal neuronal dysfunction in patients with type 2 DM. While no difference was observed in the primary endpoint of implicit time on mfERG when the whole population was analyzed together, brimonidine and somatostatin were associated with preserved neuronal function relative to the placebo group among the subset of participants with neuronal dysfunction at baseline [68].

## Does Neurodegeneration Precede Vascular Changes?

While considering the early retinal dysfunction that occurs in DM and the potential for neuroprotective strategies in the management of diabetic retinal disease, the question still remains whether retinal neurodegeneration truly precedes vascular pathology. In the HFD mouse model, ERG changes including decreased OP latency indicating bipolar cell dysfunction and pattern ERG deficits indicating that RGC dysfunction are present after 6 months of HFD feeding, preceding the onset of vascular leakage at 12 months [34, 59]. In the STZ mouse model, thinning of the GCL/IPL complex has been described as early as 6 weeks with decreased RGC density at 20 weeks, a time point when significant pericyte dropout was not detected [24].

While most animal models of DR support the argument for diabetic retinal neurodegeneration preceding retinal vascular pathology, the evidence from diabetic patients is less clear in part due to improvements in vascular imaging. Many studies have established that thinning of the inner retina (GCL, IPL) as assessed by OCT is present in patients with diabetes prior to the diagnosis of clinical DR, consistent with experimental models [4, 5]. Progression of clinical DR has been associated not only with rate of inner retinal thinning over time but also with baseline GCL + IPL thickness in patients with T2DM [20••, 69]. Areas of delayed implicit time on mfERG also predict areas of subsequent DR progression, further suggesting that neuronal manifestations may precede and potentially contribute to the vascular hallmarks of DR [19]. In a study of patients with T1DM and no or minimal DR at baseline, localized deficits on Humphrey visual field (24–2) testing were associated with development of DR [70].

However, the advent of OCT angiography (OCT-A) technology has allowed greater resolution of the retinal vasculature far beyond what has been detectable by clinical examination or fluorescein angiography, providing clinicians with the ability to visualize the superficial and deep capillary plexi (SCP, DCP) in a noninvasive manner. OCT-A and other newer imaging modalities, including swept-source OCT imaging which provides penetration into the choroid, have demonstrated preclinical retinal vascular and choroidal changes. Using OCT-A, several abnormal vascular parameters have been described in diabetic patients without DR, including decreased vascular density of the SCP and DCP and increased area of the foveal avascular zone (FAZ) [71, 72]. In a recent prospective study of patients with DM without DR (17 patients with type 1 DM, 17 patients with type 2 DM), Forte et al. demonstrated decreased vessel density in the central 1 mm (1) in the SCP in both type 1 and type 2 DM and (2) in the DCP in those with type 2 DM compared with the control group, as well as increased FAZ area in type 2 DM [73]. An independent observational case–control study of 60 eyes from diabetic patients without DR similarly identified reduced vessel density in the SCP and DCP along with visual acuity impairment measured by central vision analyzer compared with controls, but did not find any differences in FAZ [74]. Using swept-source OCT-A, both type 1 DM and type 2 DM have also been associated with flow voids in the choriocapillaris prior to clinical DR in some studies [73], while another analysis of type 1 DM patients without DR found only differences in SCP density with no changes in the choriocapillaris flow or FAZ area compared with controls [75]. Interestingly, Goudot et al. did not identify differences in any retinal capillary parameters in 22 eyes from patients with type 1 or type 2 DM without retinopathy compared with controls using OCT-A [76]. The variable findings regarding retinal capillary measurements in diabetic patients without DR using OCT-A perhaps reflects differences in the patient populations, imaging instruments employed, or analysis methods, and underscores the need for improved standardization when using this newer imaging modality. Emerging additional OCT-A analysis techniques to derive quantitative measures of vessel diameter and tortuosity have also been used to demonstrate early microvascular changes in the diabetic retina [77].

Overall, though, findings from OCT-A have raised into question whether vascular or neuronal changes occur first. A concurrent analysis of the retinal microvasculature and retinal thickness in patients with type 1 DM but no DR revealed decreased vessel density in the DCP with no difference in RNFL and GCL thickness compared with controls [78]. On the other hand, a similar concurrent analysis among patients with type 1 DM but no DR demonstrated retinal thinning without changes in vessel density on OCT-A [76]. It is very likely that the early neuronal and vascular changes in the diabetic retina are tightly coupled and dissecting the timeline of onset may continue to prove challenging. Indeed, a prospective longitudinal study of patients with type 2 diabetes demonstrated progressive decrease in the superficial vascular density along with thinning of the GCL and IPL over a two-year period, suggesting that these changes occur in parallel [79•]. Kim et al. also described a similar association between microvascular changes and inner retinal thinning in a retrospective analysis of 40 diabetic eyes without DR, highlighting the importance of neurovascular coupling in the retina [80].

## Conclusions

Studies of patients with DM prior to the onset of DR using newer more sensitive technology to assess retinal architecture, retinal capillary parameters, and visual function continue to reveal the complexity of diabetic retinal disease and expand our understanding of how diabetes affects the retina. Whereas the clinical definition, diagnosis, and staging of DR as well as current medical treatment options reflect the long-held paradigm of DR as a disease of the retinal vasculature, it is very clear now that inner retinal thinning (of the RNFL and GCL, and possibly the IPL) occurs in the eyes of patients with DM prior to clinical DR, suggesting that diabetic retinal neurodegeneration represents an important, and possibly independent, manifestation of diabetic retinal disease. Animal models and studies of intraocular fluid from diabetic eyes have implicated oxidative stress, hyperglycemia-related production of AGEs, glutamate excitotoxicity, and inflammation as potential underlying molecular mechanisms for retinal neuronal loss and dysfunction in diabetes and targeting these pathways or exogenous administration of neurotrophic growth factors may provide strategies for neuroprotection. OCT-A has allowed detailed analyses of the retinal capillary networks and indicated that preclinical vascular changes also occur in diabetic eyes, and whether these vascular abnormalities precede, follow, or coincide with inner retinal neuronal degeneration remains an open question. Regardless, the current diagnostic criteria for DR do not account for these early “pre-clinical” retinal changes that not only affect visual function but may contribute to subsequent DR progression, and an updated staging system for diabetic retinal disease (DRD) which incorporates retinal neuronal, vascular, and visual function deficits has been proposed [81••]. Recognition and understanding of diabetic retinal neurodegeneration may provide opportunities for earlier detection and novel strategies for intervention in patients with diabetes to ultimately reduce the incidence of blindness from DR.

## Funding

This work was supported by grants to M.M.S. from the National Eye Institute (K08 EY029766) and the Research to Prevent Blindness foundation (CareerDevelopment Award).
